# Partner relationship satisfaction and maternal emotional distress in early pregnancy

**DOI:** 10.1186/1471-2458-11-161

**Published:** 2011-03-14

**Authors:** Gun-Mette B Røsand, Kari Slinning, Malin Eberhard-Gran, Espen Røysamb, Kristian Tambs

**Affiliations:** 1 Norwegian Institute of Public Health, Division of Mental Health, PO Box 4404 Nydalen, N-0403 Oslo, Norway; 2 National Network for Infant Mental Health, Centre for Child and Adolescent Mental Health, Eastern and Southern Norway; 3 Health Services Research Centre, Akershus University Hospital, Norway; 4 University of Oslo, Department of Psychology, Norway

## Abstract

**Background:**

Recognition of maternal emotional distress during pregnancy and the identification of risk factors for this distress are of considerable clinical- and public health importance. The mental health of the mother is important both for herself, and for the physical and psychological health of her children and the welfare of the family. The first aim of the present study was to identify risk factors for maternal emotional distress during pregnancy with special focus on partner relationship satisfaction. The second aim was to assess interaction effects between relationship satisfaction and the main predictors.

**Methods:**

Pregnant women enrolled in the Norwegian Mother and Child Cohort Study (n = 51,558) completed a questionnaire with questions about maternal emotional distress, relationship satisfaction, and other risk factors. Associations between 37 predictor variables and emotional distress were estimated by multiple linear regression analysis.

**Results:**

Relationship dissatisfaction was the strongest predictor of maternal emotional distress (β = 0.25). Other predictors were dissatisfaction at work (β = 0.11), somatic disease (β = 0.11), work related stress (β = 0.10) and maternal alcohol problems in the preceding year (β = 0.09). Relationship satisfaction appeared to buffer the effects of frequent moving, somatic disease, maternal smoking, family income, irregular working hours, dissatisfaction at work, work stress, and mother's sick leave (*P *< 0.05).

**Conclusions:**

Dissatisfaction with the partner relationship is a significant predictor of maternal emotional distress in pregnancy. A good partner relationship can have a protective effect against some stressors.

## Background

Becoming a parent or expanding the family with a new child is an important life event that involves not only the pregnant woman, but also the partner and the extended family. For most women, pregnancy is a happy experience associated with positive expectations, but worries and concerns also increase. Many women feel vulnerable during pregnancy. They become more dependent upon their partner and support from family and friends, especially other women becomes more important during this time [1-3]. Previous studies on pregnant women's mental health have examined both specific aspects such as depression, and broader concepts such as emotional distress. Recent research suggests that antenatal depression is as prevalent as postpartum depression (PPD) [4-6]. A review published in 2004 based on 21 studies (19,284 respondents), found that the prevalence of depression during pregnancy was 7.4%, 12.8% and 12.0% during the first, second and third trimester respectively [2]. The severity and nature of the depressed mood does not seem to differ significantly pre and post birth [5].

Recognition of maternal emotional distress during pregnancy and the identification of risk factors for such distress are of considerable clinical importance because mental health problems can affect both the foetus and the mother. Anxiety and depression during pregnancy have been associated with poor pregnancy outcomes, such as preterm delivery, low infant birth weight, and small-for-gestational-age infants [2,7]. In addition, depressive symptoms during pregnancy are a significant predictor of postnatal depression [8] and breastfeeding status at six months postpartum [9]. One longitudinal study showed that antenatal depression bore a stronger relationship with negative child outcomes measured in the early school years than did maternal depression at any time point postpartum [10].

Relative to the numerous studies on risk factors for PPD, few studies have reported results on the risk factors for anxiety and depression symptoms during pregnancy. Some investigations have shown that the risk for depression (or, in some studies, poor global mental health) during pregnancy is associated with poor relationship with the partner [11,12], low or absent support from the partner [11,13], low support from extended family/friends [11,14], demographic factors such as low education [11], single status [11,14,15], financial hardship [11,16], young maternal age [12,17], many children in the household [18], high life stress [11,13], obstetric factors like first pregnancy, a past history of abortion [19], unintended pregnancy [11,20], poor pregnancy outcomes [16] and a history of depression [11,16,20]. A few studies have reported a significant effect of smoking [11,21], sleep quality [22] and poor somatic health [14,17] on depression symptoms in early pregnancy.

Most of these studies suffered from small sample sizes, and in some of them, the study population comprised of financially impoverished women [13,15]. Many of the studies are relatively old. One exception is a recent Swedish study of 3011 pregnant women, which demonstrated that lack of support from the partner, more than two stressful life events in the year prior to the pregnancy and having a native language other than Swedish were the most important predictors for depression in early pregnancy [23].

From a prevention perspective, it is important to explore possible protective factors against emotional distress in pregnancy. In accordance with the buffer hypothesis [24], some factors may help to protect against severe effects of certain strains. It has been documented that social support is an important protective factor regarding the individual's ability to handle stressful events and recovery after such events [25-27]. Thus, some types of stress will probably be tolerable for women who have good social support and high partner relationship satisfaction. Such buffer effects are usually tested as interaction effects between risk and protective factors.

To our knowledge, no other large-scale study has included such a large number of risk factors for maternal emotional distress in pregnancy as does ours. Herein, we report the results of a study consisting of 51,558 pregnant women in Norway that focused on factors related to the partner, family, and organizing everyday life. The first aim of the study was to identify risk factors for maternal emotional distress in pregnancy. We explored the contribution of 37 potential risk factors including relationship satisfaction, demographic characteristics, work-related stress, somatic diseases, negative life events and daily strain factors. The effect sizes for these risk factors were estimated. Our main hypothesis was that relationship satisfaction would be of particular importance for the women in our sample. The second aim was to assess whether high relationship satisfaction protects against severe effects of certain stressful events. We hypothesised that relationship satisfaction would protect against severe effects of certain risk factors.

## Methods

### Participants

The present study is part of the population-based Norwegian Mother and Child Cohort Study (MoBa) conducted at the Norwegian Institute of Public Health. Data collection began in 1999. MoBa aimed to enrol 100,000 pregnancies. This goal was reached in the fall of 2008. All hospitals and maternity units in Norway with more than 100 births annually were included [28]. Mothers undergoing their first routine prenatal ultrasound examination, performed at gestation week 17-18, were invited to participate in the study. 45% of the women consented to participate; 43.8% of the invited women completed the first questionnaire [28]. The assessment points in the cohort study are the 17^th ^and the 30^th ^gestational week and 6, 18, and 36 months post partum (t1-t5). Further follow ups are planned. The sample has been found to be slightly biased with regard to some demographic variables. Some groups were underrepresented, including the youngest women (< 25 years), women that lived alone, mothers with more than two previous births, and women with previous stillbirths [29]. The MoBa participants may also be somewhat more educated than the general Norwegian population [30]. Nevertheless, the sample was not found to be more than minimally biased in terms of associations between variables [29]. Sample statistics and age and sex matched population statistics on selected demographic variables are shown in Table 1. The population statistics shown in the table were taken from the net-services of Statistics Norway [31] and the Medical Birth Registry of Norway [32]. Note that the statistics on unemployment are not strictly comparable, because some pregnant women may choose not to work during pregnancy. The sample has been described in more detail elsewhere [28,29].

**Table 1:** Descriptive statistics for MoBa sample vs population

	MoBa sample		**Population**^**A**^	
		
	Proportion	Mean (SD)	Proportion	Mean (SD)
Parity				
0	43.7%		40.6%	
1	36.0%		35.6%	
2 +	20.3%		23.8%	
Age		29.9 (4.5)		29.4 (5.0)
Single^B^	2.7%		6.1%	
Education				
University, lower degree	38.9%		28.1%	
University, higher degree	17.5%		10.3%	
Unemployment	4.6%		1.1% ^C^	

^A ^Population statistics on parity, maternal age and being single for the study period (1999-2005) from Medical Birth Registry of Norway. Statistics on education and unemployment from Statistics Norway for age matched women in general (pregnant and non-pregnant) in the study period.

^B ^Separated, divorced, widowed (vs. partnered)

^C ^Observation period 2006-2009

The current study had access to the first wave of participants including 51,558 women who had returned the questionnaires filled in during the 17^th ^week of gestation. As expected, due to the large number of questions included in the questionnaire, some items had not been answered. Therefore, we chose to impute values for missing scores according to specific criteria (see below). Following the imputation procedures, 49,425 women with complete datasets were included in the analyses. The study was approved by the Regional Committee for Medical Research Ethics and the Norwegian Data Inspectorate.

### Measures

#### Main outcome variable

Maternal emotional distress was measured using a short version of the Hopkins Symptom Checklist (SCL-25) [33]. The SCL is a self-administered instrument designed to measure symptoms of anxiety and depression [34]. The five item version (SCL-5) correlates 0.92 with the original version [35]. SCL-5 has shown to have good psychometric properties [34,36]. The five anxiety and depression items is treated as a global measure of mental health, hereafter termed emotional distress. The SCL-5 [35] consisted of the following items: "Have you been bothered by any of the following during the last two weeks: 1) Feeling fearful; 2) Nervousness or shakiness inside; 3) Feeling hopeless about the future; 4) Feeling blue; 5) Worrying too much about things?". The response categories range were 1 = not bothered, 2 = a little bothered, 3 = quite bothered, and 4 = very bothered. The Cronbach alpha reliability for the SCL-5 was demonstrated to be 0.80. The SCL-scores were highly skewed with a tail to the right (mean = 1.26, SD = 0.39, skewness = 2.45, kurtosis = 8.21), and were ln-transformed to obtain closer to normal distribution. The dependent variable was standardized before inclusion in the analyses.

#### Partner relationship

To measure partner relationship satisfaction, we used the 10-item Relationship Satisfaction (RS) scale (Røysamb, E, Vittersø, J, Tambs, K: *In Progress*, 2010) developed within MoBa, based on typical items used in previously developed scales [37,38]. The RS scale has shown good psychometric properties, high structural and predictive validity, and correlates 0.92 with The Quality of Marriage Index [39]. The scale contains 10 items, such as "I am satisfied with the relationship with my partner" and "My husband/partner and I have a close relationship". The response categories ranged from 1 (strongly agree) to 6 (strongly disagree). An index of overall relationship satisfaction based on 10 items was computed as an average score across items. Cronbach's alpha for the RS scale was 0.91.

#### Sociodemographic variables

The sociodemographic variables included total family income (measured using the combined income for the mother and father), maternal age, the mother's and father's educational level (six categories from public school to >4 years at university/college). Three mutually exclusive items measuring employment situation were included and entered as dummy variables (scored no = 0, yes = 1): unemployed (disability retirement or out of work), student/military and staying at home (working at home). Paid employment was the reference category. A dichotomous variable indicating single motherhood was also included in the analyses (scored 1 if they were both unmarried, did not live with a partner, and did not respond on any item of the RS scale).

Prior to conducting the multiple regression analyses, one-way analyses of variance (ANOVAs) were performed to test for curvilinear trends. A strong curvilinear effect was found for maternal age, in which teenage mothers were clearly more distressed, and a group from 20 to 24 years somewhat more distressed, than older mothers. After 24, there was no observed age gradient. Age was therefore entered as two dummy variables in the regression analyses: age younger than 20 years, and age 20-24 years. Age older than 24 years was the reference category.

#### Variables related to social network and support

Six items on type of household were collapsed into three dummy variables: living in a nuclear family, living with extended family, and living with others. Living alone was the reference category. Social support was measured by two items. The first question covered the number of supportive persons in addition to their partner: "Do you have anyone other than your husband/partner whom you can ask for advice in a difficult situation?" There were three response categories: "no", "yes 1-2 people", "yes more than 2 people". The second question measured the frequency of contact with extended family and friends: "How often do you meet or talk on the telephone with your family (other than those you live with) or close friends?" There were three response categories: "once per month or less", "2-8 times per month", and "more than twice a week". We also included one variable dealing with "housing situation". This was a dichotomous measure indicating whether the family lived in an apartment block versus in a house.

#### Somatic diseases

The somatic indicator was based on a 46 item checklist covering seven different groups of diseases: asthma/allergy/eczema, diabetes, cardiovascular disease/high blood pressure/hyperthyroidism/hypothyroidism, gastrointestinal disease, muscular/skeletal/articular disease, gynaecological/urinary/kidney disease, and "other disease". The respondents reported whether or not they had experienced problems in these areas before or during pregnancy.

Seven summative indices were created using the sum scores for each of the seven groups of diseases. The seven indices were entered into a regression analyses with maternal mental distress as a dependent variable. A general index based on the seven disease group scores was then generated to estimate the overall effect of somatic disease. The scores for each separate disease group were weighted by their respective regression coefficients estimated in the initial analyses and then summed. This procedure maximizes the predictive power of the global somatic indicator for mental health. Replacing the original seven somatic items with this index in the principal multivariate analysis of predictors of mental health essentially leaves the variance explained by somatic diseases unchanged. The purpose of collapsing these predictors was to obtain one single estimate of the total effect of all somatic diseases. The somatic disease indicator was standardized before inclusion in the analyses.

#### Life strain

We included two dichotomous variables on the respondent's working hours: shift work and irregular work hours. Regular working hours was the reference category. In addition, information about present sick leave was included. Working conditions were measured by a 16 item scale used in previous studies [40]. A factor analysis with an oblique rotation showed that a five-factor solution yielded well-defined dimensions. The dimensions were: work stress, physically demanding work, noisy work, lack of control and dissatisfaction at work. Five summative indices were created using these dimensions. Cronbach's alpha was 0.75 for work stress, 0.80 for physically demanding work, 0.80 for noisy work, 0.52 for lack of control and 0.69 for dissatisfaction at work.

One variable that specified the number of children without access to kindergarten was included. Frequency analyses showed that only 1% of the respondents had more than one child without access to kindergarten. Therefore, this variable was recoded into a dichotomous measure (0 or ≥1).

#### Life events

The questionnaire only included three types of life events, namely frequent moving, physical violence, and sexual pressure/violence. The moving item was treated as a continuous variable with five categories from 0 to ≥4.

Two variables covered current and previous experiences of violence, and both were measured using the following question, adapted from the Abuse Assessment Screen [41]: "Have you ever in your adult life been slapped, hit, kicked, or bothered physically in any way?" Responses were categorized as 1 = no, 2 = do not remember, 3 = yes. "Current violence experience" was registered if the respondents had experienced violence during the current pregnancy, and "previous violence experience" if they had experienced violence during the six months preceding pregnancy. The last two variables about sexual pressure (referring to the last six months preceding pregnancy and the current pregnancy, respectively) asked whether the respondents had been coerced or forced to have sexual intercourse. The response categories were 1 = never, 2 = yes, coerced, 3 = yes, forced, 4 = yes, raped.

#### Maternal smoking

A dichotomous variable measuring smoking during the current pregnancy was included (smoking daily or sometimes = 1, never = 0).

#### Maternal alcohol problems

Two indices dealing with alcohol (ab)use were constructed. Five questions from Rutgers Alcohol Problems Index [42] were included in the questionnaire, and were used to construct a summative index for alcohol *problems: *"Have you ever experienced any of the following problems during the last year in relation to your alcohol consumption: 1) Argued with or had negative feelings for a family member; 2) suddenly found yourself somewhere without knowing how you got there; 3) Been absent from work or school; 4) Fainted or passed out suddenly; 5) Had a sad period". The response categories were 1 = never, 2 = once, 3 = several times. The variables were standardized before inclusion in the index. The general alcohol problem index was generated in order to estimate the overall effect of alcohol problems the last year.

A summative index measuring alcohol *consumption *during the last three months before pregnancy was calculated using four questions: "Did you drink 5 alcohol units at least once during the last 3 months before pregnancy?"; "How many units of alcohol do you usually drink when you consume alcohol?"; "How often did you consume alcohol in the 3 months before you became pregnant?"; "How many units of alcohol do you have to drink before you feel any effect?" (rated on Likert-type scales). The first three questions were derived from The Alcohol Use Disorders Identification Test [43] and modified for use in MoBa. Cronbach's alpha was 0.53 for the alcohol problems-scale, and 0.69 for the alcohol consumption scale.

#### Lifetime depression

Information on history of depression was obtained using an index based on DSM-III criteria developed by Kendler [44]. This index contained five questions about symptoms of depression. The respondents reported whether they had ever experienced any of the five types of depressive symptoms lasting at least two weeks, and whether three or more symptoms had been present simultaneously. An ordinal measure was generated in which the response categories ranged from 0 (0 or 1 depressive symptom) to 4 (5 symptoms, at least 3 at the same time).

### Treatment of missing values

#### Missing replacement

Including participants for whom data are partially missing, increases the power of the analyses. In general, the missing rates were low. For the continuous variables consisting of single items, the mean proportion of missing values was 3.3%, ranging from 0.5% to 5.9%. A substantial proportion of the mothers were not employed, and some employed mothers did not complete all items on working conditions, giving rates of missing data ranging from 11.1% to 15.7% on the five indices of working conditions. For the other variables used in the analyses, the frequency of missing values varied between 0% and 10.9% with an average of 3.6%. Maternal alcohol consumption, sexual pressure and violence experiences were the variables with highest missing rates.

We used SPSS MVA, Expectation Maximization [45] to impute values for missing scores on the continuously distributed SCL-5 and RS scales. Imputed values were generated when respondents already had reported values for more than half of the items on SCL-5, and at least half of the items on the RS scale. For single mothers, we carried out a mean substitution for the "overall" RS score.

For variables not included as items in a psychometric instrument, or where highly correlated variables suitable for predictive imputation were not available, missing values were replaced by sample means. Missing data on the indices measuring working conditions were substituted by sample mean values. In addition, we created two dummy variables, one indicating a moderate number (1-2) of missing values on the working condition items and one indicating a high number (3-5) of missing values. These variables tested whether women with incomplete data on working conditions (unemployed women and the incomplete responders), deviated from the remaining sample regarding mental distress. Missing values on the alcohol consumption and alcohol problem indices were replaced by sample means.

For the social support variables, we replaced missing values with the median category. The missing value for "housing situation" was replaced with a score of 1, indicating that the family lived in an apartment block.

Non-response on income and education may be highly non-random in terms of an upwards selection, therefore mean substitution may not be very suitable. We used maternal age and maternal and paternal income and education, which are all inter-correlated, to calculate suitable constants by which missing values were replaced. For instance mothers with missing values on income on average reported lower values than sample means on maternal age and education and paternal income and education. These mean values for income non-responders compared to the mean values for income responders were used to calculate the most suitable constant for replacing missing maternal income. For single mothers, we replaced a missing paternal income with zero. Child maintenance from the father was already included in single mothers' reported income. After replacement of missing values all but 2133 cases (4.1%) had complete data on all variables included in the analyses, resulting in a net sample size of 49,425.

### Statistical analyses

The 37 selected predictors of maternal emotional distress (besides of the two dummy variables indicating missing values) were examined by using multiple regression analyses. Preliminary one-way ANOVAs were used to test for possible curvilinear trends. Whenever nonlinear trends between predictor variables and emotional distress were detected, the predictor was grouped and entered into the regression analysis as two or more dummy variables. Interaction effects were tested in separate regression analyses together with all the predictors, one interaction term at a time.

We did not enter lifetime depression in the main analyses, due to high overlap with the dependent variable. Adjusting for this variable, which could be considered a "baseline" measure, could skew the scope of the study toward examining change in mental health as a result of factors related to pregnancy. Nevertheless, associations between history of depression and pregnancy-related and postpartum depression have been established in other studies [46-48]. Therefore, we conducted supplementary regression analyses in which lifetime depression was entered together with all the other variables, to measure the effect on maternal emotional distress.

## Results

### Effect of independent variables

Prior to the multivariate analyses we examined the statistical relationships between all explanatory variables. In total, three correlations exceeded 0.40, those between living with "other persons" and living in an extended family (r = 0.83), being single and living in a nuclear family (r = -0.44), and mother's and father's education (r = 0.42). Twelve other correlations reached values higher than 0.30. Of special interest were possible confounding between relationship satisfaction and the remaining variables. Four variables, number of supportive persons (in addition to the partner), dissatisfaction at work, maternal alcohol problems, and living in a nuclear family, correlated with absolute values ranging from 0.10 - 0.15. All other associations between the relationship satisfaction and the other explanatory variables were weaker than 0.10.

37 of the risk factors in the analyses were associated with maternal emotional distress *(P *< 0.001). Analyses revealed that most of the risk factors (26 of 37) were uniquely *(P *< 0.001) associated with maternal emotional distress (SCL-5 score). Table 2 shows the 26 risk factors that had a significant unique effect on maternal emotional distress. Only significant results *(P *< 0.001) are included in the table, whereas those variables not reaching significance are listed below the table. The table also shows unadjusted (crude) regression coefficients. SCL-5 was standardized; therefore the non-standardized regression coefficients (adjusted and crude b) indicate the expected difference in the standard deviation in SCL-5, per unit of difference in the predictor.

**Table 2:** Effect of various risk factors on the level of maternal emotional distress (scl-5 score) in gestational week 17 among 49,425 pregnant women.

Risk factor	Range	Mean	SD	% exposed	Crude b	Adjusted b	95% CI	Beta	Unadjusted r
*Sociodemographic variables*									
Maternal age (< 20)	0, 1		0.12	1.5	0.79	0.21	0.13, 0.28	0.02	0.09
Maternal age (20-24)	0, 1		0.32	12.0	0.28	0.06	0.03, 0.08	0.02	0.09
Single motherhood	0, 1		0.11	1.2	0.88	0.43	0.34, 0.51	0.05	0.09
Family income	0-12	6.48	2.07		-0.08	-0.03	-0.03, -0.02	-0.06	-0.16
Mother unemployed	0, 1		0.21	4.6	0.45	0.21	0.17, 0.25	0.04	0.10
Father unemployed	0, 1		0.18	3.5	0.39	0.09	0.05, 0.14	0.02	0.07
Mothers' education	0-5	3.35	1.38	17.5A	-0.09	-0.02	-0.02, -0.01	-0.02	-0.13
									
*Social network and family*									
Relationship satisfaction	1-6	5.29	0.65		-0.48	-0.38	-0.40, -0.37	-0.25	-0.31
Number of supportive persons	1-3	2.46	0.57		-0.19	-0.09	-0.10, -0.07	-0.05	-0.11
Contact frequency (family/friends)	1-3	2.79	0.43		-0.14	-0.06	-0.08, -0.04	-0.03	-0.06
									
*Life strain*									
Work stress	0-9	5.51	1.87		0.05	0.05	0.05, 0.06	0.10	0.10
Dissatisfaction at work	0-9	1.94	1.62		0.11	0.07	0.06, 0.07	0.11	0.18
Noisy work	0-6	0.79	1.31		0.07	0.02	0.01, 0.02	0.02	0.09
Lacking control over work situation	0-6	3.23	1.47		0.03	-0.01	-0.02, -0.01	-0.02	0.05
Irregular working hours	0, 1		0.33	12.1	0.25	0.11	0.08, 0.13	0.04	0.08
Mother on sick leave	0, 1		0.40	20.3	0.25	0.17	0.15, 0.19	0.07	0.10
Children without kindergarten	0, 1		0.32	11.5	-0.06	-0.07	-0.09, -0.04	-0.02	-0.02
Shift work	0, 1		0.40	20.4	-0.08	-0.08	-0.10, -0.06	-0.03	-0.03
Housing situation	0, 1		0.40	20.5	0.20	0.08	0.06, 0.10	0.03	0.08
									
*Life events *									
Frequent moving	0-4	1.16	1.17	5.3A	0.10	0.05	0.05, 0.06	0.06	0.11
Previous violence experience	1-3	1.03	0.24		0.41	0.08	0.04, 0.12	0.02	0.10
Current violence experience	1-3	1.02	0.18		0.43	0.10	0.06, 0.15	0.02	0.07
Previous sexual pressure	1-4	0.01	0.12		0.76	0.25	0.17, 0.32	0.03	0.09
									
*Health and lifestyle*									
Maternal alcohol problems last year	0-16		1.00		0.19	0.09	0.08, 0.10	0.09	0.18
Maternal smoking	0, 1		0.31	10.7	0.41	0.13	0.11, 0.16	0.04	0.12
Somatic disease	0-23		1.00		0.16	0.11	0.10, 0.11	0.11	0.16

Significance level for all the predictors in the table: p < 0.001.

Additional predictors included in the analyses with significant unique effects on scl-5 (p < 0.01):

Mother student/military, Fathers' education, Living in a nuclear family.

The following predictors had no significant unique effect on scl-5 (p > 0.01):

Mother staying at home, Physically demanding work, Father student/military, Father staying at home, Living with extended family, Living with others, Maternal alcohol consumption, Current sexual pressure/violence.

Effects of the dummy variables indicating missing values on 1-2 working condition items (NS) and indicating 3-5 missing values (b = 0.07, p < 0.001), respectively, are not included in the table.

A: frequency of extreme high score (Frequent moving: 4 or more times the last three years, Mothers' education: more than 4 years university education)

Of the 37 explanatory variables included in the analyses, relationship dissatisfaction had the strongest unique effect on maternal emotional distress (β = 0.25). Dissatisfaction at work (β = 0.11), somatic disease (β = 0.11), work stress (β = 0.10) and maternal alcohol problems in the last year (β = 0.09) were also among the strongest risk factors in the analyses.

There were few single mothers in the study sample, preventing a high β for being single mother. However, the adjusted increase in SCL scores for single mothers as compared to the remaining sample was 0.43 standard deviations. This means that being a single mother increases the risk for maternal emotional distress in pregnancy. Previous sexual pressure also had a considerable effect (b = 0.25) for the relatively few (n = 452) women who reported some degree of sexual pressure. Of those, 407 reported being coerced, 19 reported being forced and 26 women reported being raped. Comparing the groups with highest and lowest scores, the SCL-5 score was 0.75 standard deviations higher in the group with highest degree of previous sexual pressure. A strong curvilinear effect was found for mother's age. The adjusted deviations (b) were 0.21 (*P *< 0.001) for age younger than 20 years, and 0.06 (*P *< 0.001) for age 20-24 years. This means that younger age is an important risk factor for maternal emotional distress during pregnancy.

The following factors did not have unique significant effects on maternal emotional distress (all with *P *> 0.01): mother staying at home, physically demanding work, father student/military, father staying at home, living with extended family, living with others, alcohol consumption, and current sexual pressure, which given the large sample size implies that the effects are only minimally different from zero.

A significant but small effect, b = 0.07, of the dummy variable indicating "missing data on 3-5 working condition items" is not tabulated. The effect of the dummy variable indicating 1-2 blank working condition items did not reach significance.

### Model with lifetime depression as a risk factor

Information on history of depression was entered together with the variables included in the analysis of main effects. Lifetime depression was a stronger predictor of emotional distress than any other variable (β = 0.27, b = 0.21, *P *< 0.001). Relationship dissatisfaction was the second strongest predictor of maternal emotional distress in this model (β = 0.21, b = 0.33, *P *< 0.001). Thus, even with lifetime depression included in the analyses, the effect of relationship satisfaction remained strong. We also found that 23 of the other originally significant predictors (all but mothers' education, lacking control over work situation, and contact frequency) still had a significant effect (*P *< 0.001) on emotional distress.

### Interaction effects

Interaction effects were investigated for relationship satisfaction with the 10 variables with strongest unique main effects: dissatisfaction at work, work stress, somatic disease, maternal alcohol problems in the preceding year, maternal smoking, maternal unemployment, family income, irregular working hours, mother on sick leave, and frequent moving. The results are presented in Table 3.

**Table 3:** Interaction effects between relationship satisfaction and 10 predictors on maternal emotional distress; main effects (b) for various strata with low or high relationship satisfaction.

Relationship satisfaction	Frequent moving (0-4)	Somatic disease (SD scored)	Maternal smoking (0,1)	Family income (0-12)	Irregular working hours (0,1)	Dissatisfaction at work (0-9)	Work stress (0-9)	Mother on sick leave (0,1)
Low (lower 10%)	0.09	0.13	0.23	-0.02	0.13	0.08	0.06	0.18
Moderate (23%)	0.06	0.11	0.08	-0.03	0.11	0.08	0.05	0.18
Moderately high (29%)	0.05	0.10	0.11	-0.03	0.12	0.08	0.06	0.19
High (upper 38%)	0.03	0.10	0.13	-0.03	0.08	0.06	0.04	0.15
Significance, p	< 0.001	0.002	0.002	0.003	0.006	< 0.001	< 0.001	0.037

No significant interaction effect (p > 0.05) was found for 'Mother unemployed × Relationship satisfaction' and 'Maternal alcohol problems the last year × Relationship satisfaction'.

Significant interaction effects *(P <*0.05) were found between relationship satisfaction and the following eight predictors: dissatisfaction at work, work stress, somatic disease, maternal smoking, low family income, irregular working hours, mother on sick leave and frequent moving. In general, the results indicated that high relationship satisfaction protects against the negative effects of these eight risk factors.

## Discussion

To our knowledge, this is the first study to analyse risk factors for maternal emotional distress in a large cohort of pregnant women. In the current study including 49,425 pregnant women in Norway, 26 of the 37 predictors evaluated had a significant unique effect on maternal emotional distress. Relationship dissatisfaction, dissatisfaction at work, work stress, somatic disease and alcohol problems during the preceding year were among the strongest predictors. Relationship dissatisfaction had the strongest effect and explained 6.3% of the total variance. The results also show that a good relationship with partner may act as a protective factor against some stressors.

### Main effects

#### Relationship satisfaction

Our observation of a strong association between partner relationship and emotional distress is consistent with previous results regarding mental distress, anxiety and depression and with our main hypothesis. Partner relationship quality has been shown to be significantly associated with women's mental health [49,50] and with major depression [51-53] in general, and during pregnancy [6,12,19,54]. It is noteworthy that some studies [12,55] differentiate between partner support and relationship satisfaction and investigate the effect of each factors. For example, O'Hara (1986) found that women who experience depression during pregnancy reported less support from their spouses than did non-depressed women. They did not, however, differ from non-depressed subjects with regard to level of marital satisfaction, which according to the authors could be considered a broader phenomenon than partner support [55]. The differences and association between these concepts remain unclear. Relationship satisfaction and partner support are measured in different and sometimes overlapping ways in the various studies, making it difficult to compare the findings. Still, our results seem to be in line with previous results regarding the significance of partner relationship quality in pregnancy.

Most of the previous studies suffered from small sample sizes. One notable exception is a Swedish study of 3011 pregnant women [23], which showed that lack of support from the partner was the most important risk factor for depressive mood. However, partner support was measured with only one item. Our more comprehensive measure of partner relationship satisfaction, our large variety of covariates, and our large sample size ensure greater applicability of the results and more precise effect estimates than did the Swedish study.

Our findings can be interpreted in several ways. One explanation for the importance of relationship satisfaction in pregnancy may be associated with the mothers' particular vulnerability and need for support during this time. The results from one study showed that the lack of a cohabiting partner was a significant risk factor for depression during pregnancy, but not postpartum [15]. The transition to parenthood can be a critical phase and a time when a woman may benefit the most from partner support. A pregnant woman must not only carry the baby to term safely, but also accept the sacrifices that motherhood demands. She must ensure the acceptance of the child by the family, develop an attachment to the baby within, and prepare for the birth. She must also adjust to the alteration in her physical appearance and develop a somewhat different relationship with the father of the child. However, studies of depression postpartum have also found an association between depression and having no partner [56,57], or experiencing a low relationship satisfaction [56,58].

Having a child is a mutual project for the couple. Previous research has shown that most pregnant women receive their primary social support from their partners and their own mothers [59,60]. Other studies have demonstrated that the single most important source of social support for expectant mothers is their partners [61]. Hobfoll (1986) demonstrated that social support could be gleaned from other sources, but that partner support was especially critical during pregnancy and delivery [62]. This could explain the strong negative effect of single motherhood observed in the current study.

Results from a study of Mexican immigrant women in Los Angeles suggested that spousal support might not have the same significance for all cultural groups. These women experienced less prenatal and postnatal anxiety if they received support from friends and family, but there was no evidence of a relationship between anxiety and lack of support from the spouse [63]. The strong effect of partner relationship observed in the current study probably reflects the somewhat weaker position of extended families and the somewhat stronger position of nuclear families in Norway than in most other countries. However, an association between partner relationship quality and depression in the postpartum period is demonstrated in Asian cultures as well [64].

In cross-sectional studies like ours, any conclusions drawn about causal direction are tentative. Thus, it could be that high levels of maternal emotional distress contribute to relationship problems. Previous studies reported that relative to partners of non-distressed women, partners of distressed women reported significant reductions in the partnership quality during pregnancy and postpartum. They also had higher levels of anxiety and anger [12].

#### Other predictors

Another striking finding was that the respondents' working conditions seemed to be important for emotional distress during early pregnancy. Dissatisfaction at work and work stress were the second and fourth strongest predictors of emotional distress in our sample. Research has demonstrated that working conditions in pregnancy may affect pregnancy outcome [65]. To our knowledge, no previous studies have examined specific aspects of women's work situation as possible risk factors for emotional distress in pregnancy. Studies from the general population have demonstrated that the risk of depressive and anxiety disorders increases with various work characteristics, such as a high level of psychological demands, effort-reward imbalance, low decision authority at work, low job control, high job strain, and low support [66-70].

Working can be both advantageous and detrimental for mental health [69,71]. Plaisier et al. found that having a job protected against anxiety and depression for men and women without children but not for women with children [72]. Women with young children might be more susceptible to the stresses of juggling the demands of multiple roles, such as worker and parent, which could be detrimental to mental health. In our sample, 49.8% of the women had children and were also working or studying. One possible explanation for the strong effect of work-related variables is that work dissatisfaction and work stress come in addition to the existing stress of multiple roles for a large number of the respondents.

Our findings indicate that somatic disease either before or during pregnancy had a considerable negative effect on maternal emotional distress in pregnancy. This is in line with previous research demonstrating that prenatal depression and anxiety is associated with more frequent somatic symptoms [73,74]. An association between maternal somatic health and depression has also been shown in the postpartum period [75], and among mothers of toddlers and pre-school children [76].

Alcohol disorders have often been reported to be a risk factor for depressive disorders [77]. However, in a recent study based on a Swedish community sample, alcohol disorders represented a risk factor only for males [78]. Alcohol consumption has been clearly associated with adverse/severe and sometimes long-lasting consequences for the child, even when consumed in small to moderate quantities during pregnancy [1]. Therefore, alcohol problems might affect mental health among pregnant women even stronger than among women in general. The results from the current study showed that having alcohol problems during the year preceding pregnancy was a significant risk factor for maternal emotional distress during pregnancy. To our knowledge, only a few studies have examined the effect of maternal alcohol problems on emotional distress in pregnant women. One Finnish study showed a significant association (OR = 3.4, *P *< 0.001) between substance dependency and maternal depression in a sample of 391 women who were 14-37 weeks pregnant [79].

Previous sexual pressure was reported by few women in our sample but showed a considerable effect for the women affected. Comparing the groups with highest and lowest scores, the SCL-5 score was 0.75 standard deviations higher in the group with the highest degree of previous sexual pressure as compared to the women who reported no sexual pressure. In contrast, a previous Norwegian study assessed depression in 2730 post-partum and non-postpartum women, and found no evidence of increased depression scores among women having been pressed or forced to intercourse. This was the case for both postpartum and non-postpartum women [46]. Under-reporting of such experiences might hinder detection of risk effects in any but very large samples.

Being a single mother increased the risk for emotional distress in pregnancy. The Scandinavian countries are characterised by reasonable social benefit for lone mothers, long term paid parental leave and good access to childcare, which are all important factors for parent's well-being [76], and maybe single parents in particular. Nevertheless, being single seems to represent a considerable strain for pregnant women.

Also noteworthy is the absence of effects from certain factors, such as alcohol consumption. Some previous studies have investigated the effect of alcohol use on depression in pregnancy, and the findings are inconsistent [11]. The results from the current study show that alcohol problems have a significant unique effect on emotional distress, while alcohol consumption has not. Given the large sample size of this study, the absence of effects is informative.

### Interaction effects

The interaction between mental health, social network and stress is complex. In accordance with our hypothesis, relationship satisfaction seemed to act as a buffer [24,26] for some risk factors, like work stress, dissatisfaction at work, and frequent moving. When women reported having a good relationship, they seemed better able to cope with such strains without experiencing increased emotional distress. To our knowledge, only a few studies have investigated interaction effects between relationship satisfaction and other factors on maternal emotional distress in pregnancy. One study examined the effect of life stress and social support (from partner, relatives and friends) on anxiety in a sample of 190 low-income women. In addition to the main effects of social support, their findings indicated a significant stress-buffering effect from social support [13]. Results from the current study further contribute to this body of evidence by demonstrating the buffering effects of relationship satisfaction.

### Strengths and limitations

High statistical power and precise estimates are the most important strengths of this study. Small effects and even negative results are still highly informative due to the narrow confidence intervals. The large number and variety of predictor variables are also important advantages.

Nonetheless, our findings must be interpreted carefully due to several limitations. First, in cross-sectional studies, the data are not informative regarding causal directions. Although we find it likely that a good partner relationship protects against poor mental health, we cannot rule out the reverse causal pathway [80]. Second, there may be response biases that cause spurious correlations between self-reported predictor and outcome variables. Third, the validity and reliability of a brief self-report scale such as the current outcome measure might be less than optimal. However, a reasonably good validity was demonstrated in a recent study, comparing a dichotomised SCL-5 with an "any disorder" variable based on the Composite International Diagnostic Interview CIDI [81]. This variable was scored '1' if any anxiety or depression disorders were present, else '0'. Furthermore, the results indicated that all this statistical correspondence was due to genes that coded both for the SCL scores and for the internalizing disorders measured with the CIDI [82]. The first and second limitation could have led to inflated estimates, whereas the third could have deflated them. Fourth, 43.8% of the invited women completed the first questionnaire. Although this response rate is low, it is not uncommon in large epidemiologic studies and does not necessarily imply a biased sample [83]. In addition, while preventing reliable estimation of occurrence of mental health problems, a moderate sample selection is not expected to affect results from analytic epidemiology dramatically [83]. Significant mean differences in prevalence estimates between the cohort participants and the total population have been found for certain variables. Despite this, no statistically significant differences in exposure-outcome associations are found [29]. This indicates there is little reason to consider the selection bias a threat to the generalizability of our results showing associations between variables, like relationship satisfaction and emotional distress.

## Conclusion

Our findings demonstrate the importance of partner relationship quality, working conditions, alcohol problems, and somatic disease as predictors of maternal emotional distress in early pregnancy. Even though perinatal depression is a well known clinical phenomenon, women at risk are rarely recognised by health professionals [6,47,84]. Traditionally, medical and midwife practitioners have focused on the physical health of the mother and the foetus. Considerably less attention has been given to emotional and psychological issues [12]. The mental health of the mother is important not only for herself, but also for the physical and psychological health of her children and the welfare of the family. Hence, failure to recognize and assist women who suffer from emotional distress during pregnancy is failure to address a major public health problem [85].

Our results suggest a need for a more comprehensive antenatal program which focuses on the foetus' development, the woman's somatic health, as well as the woman's mental well- being. The importance of a good partner relationship that consists of both emotional and practical support should be highlighted to all expecting couples. Many expecting couples participate in courses that prepare them for the delivery. These courses could therefore be extended to include topics and exercises that strengthen positive aspects of their relationship. All signs of trouble in a woman's adaptation to pregnancy should be taken seriously by obstetricians, nurse midwives, and mental health professionals. Special attention needs to be paid to the woman's relationship with her partner. Early intervention that involves both the woman and her partner may be successfully initiated in some cases to strengthen the foundation for the family's future development.

Relationship dissatisfaction was clearly associated with maternal emotional distress, and relationship satisfaction appeared protective in the presence of certain stressful events. Thus, some types of stress will probably be tolerable for women who experience a good and close relationship with their partners. Working conditions, somatic diseases, and alcohol problems should also be taken into consideration. From a prevention point of view, it is important to pay attention to women who appear to lack the protection from a good partner relationship and who simultaneously experience several risk factors.

## Competing interests

The authors declare that they have no competing interests.

## Authors' contributions

GMBR performed the statistical analyses and drafted the manuscript. All authors contributed to the study's design, preparation of the data, interpretation of results and helped to draft or critically revise the manuscript. All authors read and approved the final manuscript.

## Pre-publication history

The pre-publication history for this paper can be accessed here:

http://www.biomedcentral.com/1471-2458/11/161/prepub
